# Unaware and unpowered: evaluating patient perceptions and preferences of biosimilars in South Korea

**DOI:** 10.3389/fphar.2025.1551451

**Published:** 2025-03-14

**Authors:** Eunjung Choi, Gyeongseon Shin, SeungJin Bae

**Affiliations:** College of Pharmacy, Ewha Womans University, Seoul, Republic of Korea

**Keywords:** biosimilar, patient, South Korea, education, knowledge, attitude, uptake

## Abstract

**Objectives:**

Biosimilars offer a promising solution to challenges related to healthcare budget sustainability. However, limited patient awareness and understanding often hinder their timely adoption. This cross-sectional survey evaluates the perceptions, preferences, and experiences of South Korean patients regarding biosimilars.

**Methods:**

An anonymous, self-administered, web-based survey comprising up to 26 questions was conducted. Participants were recruited from Ewha Womans University Medical Centers from November 2023 to August 2024. The analysis focused on respondents with medical conditions typically treated with biologics, such as solid tumors, blood cancers, and autoimmune diseases.

**Results:**

Out of 133 responses, 100 were analyzed after excluding 33 individuals with irrelevant medical conditions. Among these, 66% had heard of biosimilars, primarily through the internet (28.8%, 19 out of 66). However, 55% were unfamiliar with the definition of biosimilars, and 61% did not understand the difference between generics and biosimilars. While most respondents considered biosimilars comparable to originators in terms of safety (45%) and efficacy (41%), the primary concerns were a lack of confidence in their safety (50%) and efficacy (50%). Among patients who exclusively used either originators or biosimilars, 91.7% and 95%, respectively, cited their doctors’ recommendations as the main reason for choosing their treatment.

**Conclusion:**

Patients primarily rely on doctors’ recommendations, yet their awareness and understanding of biosimilars remain limited. To enhance positive perceptions of biosimilars among patients, implementing diverse educational programs and actively involving a multidisciplinary health team is essential. Such initiatives will not only increase patient access to these treatments but also contribute to the long-term sustainability of healthcare systems by encouraging the broader adoption of biosimilars.

## 1 Introduction

Biologic drugs have become dominant pipelines in the pharmaceutical industry. Notably, five out of the top ten projected best-selling products in 2023 were biopharmaceuticals, and this figure could have been even higher if COVID-19 treatments were excluded (Brown, 2023). However, the dominance of biologic drugs in the pharmaceutical industry presents a significant challenge to the sustainability of healthcare budgets due to their high prices (Shin et al., 2024; Wu et al., 2023). The rising cost of pharmaceuticals lays a significant financial burden in many countries, potentially leading to the long-term collapse of healthcare systems. This issue related to biologics can possibly be addressed by adopting biosimilars.

Biosimilars, named for their similarity to originator biologics in terms of quality, safety, and efficacy, provide more affordable alternatives while delivering comparable clinical health outcomes (Baumgart et al., 2019; Shin et al., 2024; Wu et al., 2023). Their introduction has successfully yielded significant economic benefits in various countries (Aladul et al., 2017a; Baumgart et al., 2019; Chew et al., 2022; Matusewicz et al., 2015; Mulcahy et al., 2022).

Recognizing their potential, many countries, including South Korea, have established regulatory frameworks to ensure their quality, safety, and efficacy. In South Korea, biosimilars are regulated under the Regulations on Product Authorization and Review of Biological Products by the Ministry of Food and Drug Safety. These products must undergo comprehensive quality, non-clinical, and clinical evaluations before approval to ensure they are safe and effective alternatives to originators (Bas and Castillo, 2016; Ministry of Food and Drug Safety, 2022).

However, despite their regulatory approval and cost-saving potential, biosimilars are not as widely prescribed as originators (Sarnola et al., 2020). This trend is particularly evident in East Asian countries such as South Korea and Japan, where biosimilar uptake remains lower than in Europe (Hara et al., 2019; Kim et al., 2020; Woo et al., 2024). This failure of biosimilar market penetration can be attributed to various factors, including the absence of usage-enhancing policies, the high cost of biosimilars, and insufficient awareness among prescribing physicians and patients (D’Amico et al., 2023; Kolbe et al., 2021). Specifically, patients’ reluctance to use biosimilars was identified as a major barrier to physicians prescribing them (Beck et al., 2016).

Patient understanding of biosimilars plays a crucial role in treatment adherence (Khoo et al., 2022; Rezk and Pieper, 2018). Assessing patients’ awareness is critical for not only developing effective educational programs and materials, but also for planning strategies and implementing relevant healthcare policies (Jacobs et al., 2016; Pölkki and Prami, 2024). Given these considerations, several surveys exploring patient perceptions and attitudes towards biosimilars have been conducted. Most were predominantly done in Europe where the biosimilar market is most activated (Aladul et al., 2017b; Azevedo et al., 2018; Frantzen et al., 2019; Haghnejad et al., 2020; Kaneko et al., 2022; Macaluso et al., 2020; Peyrin-Biroulet et al., 2019; Peyrin-Biroulet et al., 2017; Van Overbeeke et al., 2017; Vandenplas et al., 2022; Waller et al., 2017), as well as in North America (Chau et al., 2019; Chew et al., 2022; Papautsky et al., 2022; Teeple et al., 2019), and Oceania (Gasteiger et al., 2021; Khoo et al., 2022).

However, in Asian countries, where doctor-patient communication tends to be more passive and one-sided due to cultural context (Matusitz and Spear, 2015), there has been a notable lack of studies investigating patient perceptions. A recent study across six Asian countries assessed patients’ perceptions of biosimilars, identifying cost savings and coverage support as key benefits from biosimilars (Thongpooswan et al., 2024). Additionally, a survey conducted in China revealed that about half of the respondents were unaware of the precise definition of biosimilar (Hu et al., 2022). Both studies predominantly involved healthcare providers, with only 17.4% and 15.4% of the research population being patients, respectively. This trend indicates patients’ perspectives have often been overlooked compared to those of physicians, despite their potential to significantly influence biosimilar market dynamics. Contrary to the main focus on healthcare providers in previous studies, this survey specifically aims to explore patients’ general perception, preferences, and satisfaction regarding biosimilars, which could serve as a foundation for shaping biosimilar-related policies.

## 2 Materials and methods

### 2.1 Target population

Survey respondents were recruited from Ewha Womans University Mokdong and Seoul hospitals, and recruitment flyers with a QR code linked to the survey were displayed on bulletin boards in disease-specific centers frequently prescribing biologics. These centers included the cancer outpatient center, the inflammatory bowel disease center, and the rheumatology center, etc. The survey was conducted from November 2023 to August 2024. The target population comprised a convenience sample of patients diagnosed with conditions commonly treated with biologics, such as solid tumors, blood cancers, or chronic autoimmune disorders. To ensure voluntary participation in the survey, respondents must be at least 19 years old and proficient in Korean to comprehend the study details and provide informed consent. Those not meeting these criteria were excluded from the analysis.

### 2.2 Survey design

An anonymous, self-administered, web-based cross-sectional survey was conducted, with an estimated completion time of approximately 10 min. The survey consists of 26 interactive questions tailored to the participant’s response. The questionnaires were developed based on previous studies that evaluated patients’ knowledge and perception of biosimilars (Azevedo et al., 2018; Chau et al., 2019; Chew et al., 2022; Frantzen et al., 2019; Jacobs et al., 2016; Kaneko et al., 2022; Macaluso et al., 2020; Papautsky et al., 2022; Peyrin-Biroulet et al., 2019; Peyrin-Biroulet et al., 2017; Waller et al., 2017) and adapted to suit the circumstances in South Korea. The survey composed up to 20 pages (screens), with each containing between 1 and 5 questions. Questionnaires translated into English and survey flow chart are available in the additional file (Supplementary Figure S1). All questions were pretested for comprehensibility and validity by a representative of a Korean patient organization. This study adheres to the Checklist for Reporting Results of Internet E-Surveys (CHERRIES) guidelines (Supplementary Table S1) (Eysenbach, 2004).

As the survey was open to anyone who had access to the recruitment flyers, participants were required to log in using their Google account to prevent multiple submissions from the same individual. Upon accessing the survey link, participants were provided with an introductory page outlining the purpose of the study. Only participants who consented after reading the details could proceed to the questionnaire, and only those who answered all questions could submit their responses. Participants were able to review and modify their answers before final submission. As an incentive, a coffee voucher was provided to all participants who completed the survey.

### 2.3 Ethical Approval

This research adhered to national ethical guidelines and was approved by the Ewha Womans University Institutional Review Board (IRB ewha-202309-0028-04).

### 2.4 Statistical analyses

All categorical responses were descriptively analyzed using Excel, examining frequencies and proportions.

## 3 Results

### 3.1 Participants characteristics

133 responses were collected with a 100% completion rate, of which 100 were analyzed after excluding 33 individuals with irrelevant medical conditions for biologic use such as common cold and hypertension. The demographic characteristics of the survey respondents are presented in Table 1.

**TABLE 1:** Demographic characteristics of participants.

	N	%
Sex (N = 100)		
Female	57	57.0
Male	43	43.0
Age (N = 100)		
19–29	13	13.0
30–39	23	23.0
40–49	39	39.0
50–59	19	19.0
60+	6	6.0
Diagnosis (N = 100)		
Autoimmune disorder (rheumatism, psoriasis, Crohn’s disease, ankylosing spondylitis, etc.)	63	63.0
Solid tumor (breast cancer, lung cancer, etc.)	26	26.0
Blood cancer (lymphoma, leukemia, myeloma, etc.)	11	11.0
Date of Diagnosis (N = 100)		
Less than 1 year ago	29	29.0
1–3 years ago	27	27.0
4–6 years ago	18	18.0
7–9 years ago	8	8.0
More than 10 years ago	18	18.0
Annual Medical Expense (N = 100)^a^		
Less than $1,000 USD	28	28.0
$1,000 - less than $3,000 USD	32	32.0
$3,000 - less than $5,000 USD	16	16.0
$5,000 and above	23	23.0
Don’t know	1	1.0
Monthly Income (N = 100)^a^		
Less than $2,000 USD	4	4.0
$2,000 - less than $4,000 USD	35	35.0
$4,000 - less than $6,000 USD	23	23.0
$6,000 - less than $8,000 USD	11	11.0
$8,000 USD and above	27	27.0
Primary Treatment Hospital Location (N = 100)^b^		
Metropolitan	78	78.0
Nonmetropolitan	22	22.0

^a^ Note on Currency Conversion: For the purpose of simplification in analysis, an approximate currency conversion rate of 1,000 KRW to 1 USD has been assumed. This conversion is used solely for facilitating easier understanding and comparisons within the text and does not reflect actual exchange rates. Readers should note that this is a simplified assumption to aid quick comprehension and may not represent real-time currency values.

^b^ Note on Regional Classification: In South Korea, “Metropolitan” refers to the regions of Seoul, Incheon, and Gyeonggi. “Nonmetropolitan” includes the areas of Busan, Ulsan, Daegu, Daejeon, Gyeongnam, Gyeongbuk, Chungnam, Chungbuk, Sejong, Gwangju, Jeonnam, Jeonbuk, and Gangwon.

There were more female respondents (N = 57) than male (N = 43). The majority of respondents were in their 40s (N = 39), followed by those in 30s (N = 23) and 50s (N = 19). Regarding health conditions, the majority of respondents (N = 63) were diagnosed with autoimmune disorders—rheumatism, psoriasis, Crohn’s disease, and ankylosing spondylitis—followed by solid tumors (N = 26) and blood cancer (N = 11). The date of diagnosis with their disease varied, but less than a year ago (N = 29) and 1–3 years ago (N = 27) were the most common. More than half of the participants (N = 60) reported spending medical expenses under $ 3,000 USD per year. Of the 23 people who answered that they spend more than $5,000 USD in medical expenses annually, 34.8% (N = 8) had autoimmune disorders, and 65.2% (N = 15) had solid tumors or blood cancer. The majority (N = 55) had monthly incomes between $2,000 USD and $6,000 USD, and their primary treatment hospitals were located in metropolitan areas (N = 78).

### 3.2 Biosimilar general perceptions

Table 2 presents the general perceptions regarding biosimilars. When participants were asked if they had heard of biosimilars and their primary sources of information, 66% (N = 66) indicated they had heard of biosimilars. The primary sources of information reported were the internet (N = 19) and doctors (N = 18), which accounted for 56.1% of responses. Notably, only 4.5% of participants reported accessing biosimilar information through patient education programs or from other healthcare providers such as pharmacists and nurses. Among those who had not heard of biosimilars, half of them (17 out of 34) reported to have used or are currently using biologic injections.

**TABLE 2:** General perceptions regarding biosimilars.

	N	%
Heard of biosimilars (N = 100)		
Yes	66	66.0
No	34	34.0
Primary Source of Information, If Yes (N = 66)		
Internet	19	28.8
Doctors	18	27.3
Broadcast Media (TV, Radio, etc.)	9	13.6
Articles (Newspapers, Magazine, etc.)	7	10.6
Patient Association	4	6.1
People Around Me (Family, Friends, etc.)	4	6.1
Journals	2	3.0
Healthcare providers (pharmacists, nurses)	2	3.0
Patient education program	1	1.5
Understanding of Biosimilars Definition (N = 100)		
Very well	4	4.0
Somewhat	41	41.0
Not much	43	43.0
Not at all	12	12.0
Understanding of Difference Between Generics and Biosimilars (N = 100)		
Very well	8	8.0
Somewhat	31	31.0
Not much	45	45.0
Not at all	16	16.0
Biosimilar Price (N = 100)		
Expensive	62	62.0
Appropriate	26	26.0
Inexpensive	4	4.0
I do not know	8	8.0

Furthermore, 55% of participants were unfamiliar (including responses of “not much” and “not at all”) with the definition of biosimilars, and 61% reported not recognizing the difference between generics and biosimilars. Among those unaware of the difference between generics and biosimilars, 83.6% (51 out of 61) also did not know the definition of biosimilars. Similarly, among those who did not know the definition of biosimilars, 92.7% (51 out of 55) did not know the difference between generics and biosimilars as well. In addition, 64.7% (33 out of 51) of those unfamiliar with the definition and difference between generics and biosimilars were current or former biologics users.

Regarding the price of biosimilars, only 26% of participants considered the price to be reasonable, while 62% deemed biosimilars in South Korea to be expensive. In fact, among the 38 high-income participants with an average monthly income of $6,000 USD or above, 68.4% (N = 26) indicated that biosimilars are high-priced.

### 3.3 Concerns regarding biosimilars

Regarding the safety and efficacy of biosimilars, a significant proportion of participants believed that biosimilars and originators are equivalent in safety (45%) and efficacy (41%), as detailed in Table 3. However, about one-third of participants thought biosimilars are less safe (28%) and biosimilars are less effective compared to originators (29%). Among those who negatively evaluated biosimilars, 20 participants indicated biosimilars are worse in both safety and efficacy. A small group of participants held positive views of biosimilars. Eleven participants who rated biosimilars as safer than originators also believed they were either equivalent to or more effective than originators in terms of efficacy. Similarly, 92.3% (12 out of 13) of participants who rated biosimilars as having better efficacy than originators also evaluated their safety positively.

**TABLE 3:** Participants’ concerns regarding biosimilars.

	N	%
Safety (N = 100)		
Biosimilars are safer than originators	11	11.0
Biosimilars and originators are equally safe	45	45.0
Biosimilars are less safe than originators	28	28.0
I do not know	16	16.0
Efficacy (N = 100)		
Biosimilars have better efficacy than originators	13	13.0
Biosimilars and originators have equal efficacy	41	41.0
Biosimilars have lower efficacy than originators	29	29.0
I do not know	17	17.0
Concerns (N = 100)^a^		
No concerns	8	8.0
Not familiar with biosimilars	18	18.0
Lack of confidence in efficacy of biosimilars	50	50.0
Lack of confidence in safety of biosimilars	50	50.0
Biosimilar products are too expensive	19	19.0
Other	2	2.0

^a^ Multiple choices allowed.

While many participants considered biosimilars equivalent to originators in terms of safety and efficacy, concerns remained. Specifically, 50% of participants expressed a lack of confidence in biosimilars’ safety and efficacy. Additionally, some participants were also concerned about the high price (19%) and unfamiliarity with biosimilars (18%). Another concern, specified by two participants, was that biosimilars do not feel like “original” medications.

### 3.4 Preferences and biologics experiences

Table 4 outlines the biosimilar preferences and experiences of patients. In terms of preference, the majority of participants did not express a definitive preference for biosimilars (N = 61). Specifically, 28% were willing to use biosimilars, 11% were not willing to use biosimilars.

**TABLE 4:** Patients’ experience with biologics and their preferences towards biosimilars.

	N	%
Biosimilar preference (N = 100)		
Willing to use	28	28.0
Neutral	61	61.0
Not willing to use	11	11.0
Experience with Biologics (N = 100)		
Yes	67	67.0
No	33	33.0
Duration of Biologics Use (Biologics users only, N = 67)		
Less than 1 year	36	53.7
1–3 years	15	22.4
4 years or more	16	23.9
Type of Treatment Received (Biologics users only, N = 67)		
Have only used originators	24	35.8
Have only used biosimilars	20	29.9
Have used both originators and biosimilars	16	23.9
Unsure about type used (originators or biosimilars)	7	10.4

Additionally, when participants were asked if they have ever used biologics—including both originators and biosimilars—and the specific types of biologics they have been or are being treated with, 67% (67 out of 100) participants reported being biologics users. Approximately half of these users (36 out of 67) were new to biologics, meaning they have used biologics for less than 1 year. The types of treatment among biologics users varied: 35.8% (24 out of 67) have only used originators, 29.9% (20 out of 67) have only used biosimilars, 23.9% (16 out of 67) have used both originators and biosimilars, and 10.4% (7 out of 67) were unsure whether they had used originators or biosimilars.

**FIGURE 1:**
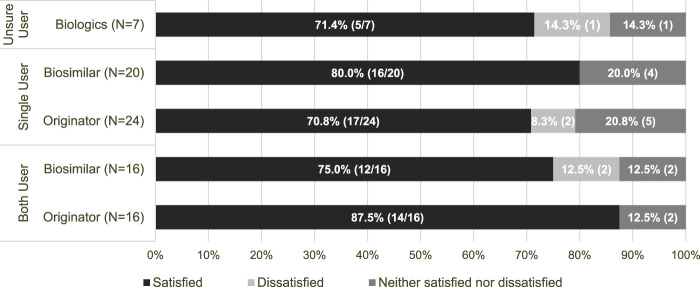
Patient satisfaction levels based on type of biologics used. Note on user classification: In this figure, “unsure users” refers to individuals who were uncertain whether they have used originators or biosimilars. “Single users” refers to individuals who have exclusively used either originators or biosimilars. “Both users” refers to individuals who have experience with both originators and biosimilars.

Participants were asked to evaluate their level of satisfaction with their biologic treatments. The detailed responses are presented in Figure 1. The majority reported high levels of satisfaction in general. Specifically, 71.4% of participants, unsure about their type of treatment, reported satisfaction with biologics. Furthermore, 80.0% (16 out of 20) of those who used only biosimilars and 70.8% (17 out of 24) of those who used originators expressed satisfaction with their respective treatment. Among those who have experience with both treatments, 75.0% (12 out of 16) were satisfied with biosimilars, and 87.5% (14 out of 16) were satisfied with originators. Overall, instances of dissatisfaction were low across all groups.

### 3.5 Decision to use biologics

As seen in Table 5, most participants who have exclusively used either originators (91.7%, 22 out of 24) or biosimilars (95.0%, 19 out of 20) indicated that the primary factor influencing their decision was the doctor’s prescription and recommendation. Other reasons for choosing originators included “previous treatment did not work” and “other healthcare providers also used originators.” A reason cited for choosing biosimilars was the belief that biosimilars are equivalent to originators.

**TABLE 5:** Factors affecting the choice between originators or biosimilars among participants who exclusively used one type.

	N	%
Factor Influencing Decision to Choose Originators (Originator users only, N = 24)		
My doctor recommended and prescribed originators	22	91.7
Previous treatment did not work	1	4.2
Other healthcare providers also used originators	1	4.2
Side effects from previous treatments	0	0.0
Originator product was easier to inject	0	0.0
Factor Influencing Decision to Choose Biosimilars (Biosimilar users only, N = 20)		
My doctor recommended and prescribed biosimilars	19	95.0
Biosimilars are equivalent to originators	1	5.0
Previous treatment did not work	0	0.0
Side effects from previous treatments	0	0.0
Biosimilars are cheaper than originators	0	0.0
Biosimilar product was easier to inject	0	0.0
Other healthcare providers also used biosimilars	0	0.0
Circumstances for Switching to Biosimilars (Originator users only, N = 24)^a^		
When my doctor recommends or prescribes biosimilars	14	58.3
When biosimilars are cheaper than originators	8	33.3
When biosimilars have proved effectiveness for my condition	6	25.0
When I experience side effects from originator	6	25.0
When biosimilars are easier to inject than originators	5	20.8
When I am dissatisfied with my current treatment	5	20.8
I would NEVER switch under any circumstances	0	0.0

Note: Percentages in this table have been rounded to one decimal place, which may result in a total that slightly exceeds 100%.

^a^ Multiple choices allowed.

Among the 24 participants who have used only originators, their responses to the question of under what circumstances they would switch to biosimilars were as follows. Most patients (58.3%, 14 out of 24) indicated that they would be willing to switch to biosimilars if their doctor prescribed or recommended biosimilars. The following most common response was if biosimilars were cheaper than originators (33.3%, 8 out of 24). Equal numbers of patients (25.0%, 6 out of 24) indicated that they would switch to biosimilars if biosimilars were proven effective for their condition or if they experienced side effects with originators. Lastly, 20.8% (5 out of 24) of patients would consider switching if they were dissatisfied with their current treatment or if biosimilars were easier to administer than originators. No participants stated that they would never switch under any circumstances.

## 4 Discussion

We found that unlike previous studies conducted in Europe and North America where the majority of had not heard of biosimilars (Peyrin-Biroulet et al., 2017; Teeple et al., 2019; Vandenplas et al., 2022), 66% of Korean patients had heard of them. However, this exposure did not directly translate into general knowledge, as over half assessed themselves not knowing the definition of biosimilars (55%) and the differences between generics and biosimilars (61%). Meanwhile, one-half of those who had not heard of biosimilars (17 out of 34) were biologics users. Considering that 67 participants were biologics users in total (as shown in Table 4), this indicates that one-fourth of biologics users were not even aware of the existence of cheaper alternatives–biosimilars—to originators. This finding aligns with prior research conducted among European patients (Peyrin-Biroulet et al., 2017). However, given that the survey questions were self-assessments and a high number of respondents did not know the difference between generics and biosimilars, it is likely that even those who claimed to know the definition of biosimilars might not realize that biosimilars are not merely generic copies of originators. The most common source of information for patients who had heard of biosimilars was the internet (19 out of 66), followed by doctors (18 out of 66). However, far fewer reported learning about biosimilars through patient education program (1.5%) and other healthcare providers like nurses and pharmacists (3.0%). This disparity underscores a significant gap in the utilization of multidisciplinary healthcare providers and patient education programs in disseminating knowledge about biosimilars. In addition, a previous study finding suggested that patients who learn about biosimilars from the internet are more concerned about switching to biosimilars and preferred originators (Gasteiger et al., 2021). In other words, self-learning from the internet should be approached with caution, as it may expose patients to misinformation about biosimilars, potentially worsening their perception of these treatments. Therefore, patient education should be implemented at the government level.

This study comprehensively investigates patient perceptions, preferences, and satisfaction regarding biosimilars within an East Asian context. Unlike previous studies in Asia, which primarily focused on both physicians and patients—with a greater emphasis on physicians (Hu et al., 2022; Thongpooswan et al., 2024)— this survey uniquely centers on patients alone. Surveys among Asian patients have been notably limited, as Asian patients have less autonomy in choosing medication, which may be due to a more passive attitude (Matusitz and Spear, 2015) or strong trust in their doctors (Mori et al., 2023). However, previous surveys of physicians and healthcare associates have shown that patient acceptance and interest in biosimilars are one of the key factors influencing physicians’ prescribing decisions (D’Amico et al., 2023; Kolbe et al., 2021). Given these considerations, this study adds significant value to the underexplored area of biosimilar research. Our study suggested that patients are unfamiliar with biosimilars, have conflicting opinions regarding their safety and efficacy, perceive biosimilars as expensive, and exhibit a neutral stance toward biosimilars. Several previous studies have indicated that patients are generally unfamiliar with biosimilars (Aladul et al., 2017b; Azevedo et al., 2018; Frantzen et al., 2019; Jacobs et al., 2016; Macaluso et al., 2020; Peyrin-Biroulet et al., 2017; Van Overbeeke et al., 2017; Vandenplas et al., 2022; Waller et al., 2017), and our findings align with these earlier studies.

Two unique tendencies were observed regarding the safety and efficacy of biosimilars. Firstly, patients exhibited a paradoxical perception: while many positively evaluated the safety (N = 45) and efficacy (N = 41) of biosimilars, the most common concerns regarding biosimilars were still focused on the safety (N = 50) and efficacy (N = 50). More specifically, among the patients who rated the safety of biosimilars as equal to that of originators, about one-third of them (35.6%, 16 out of 45) expressed concerns about biosimilar safety. Similarly, among those who rated the efficacy of biosimilars as equivalent to originators, about half of them (48.8%, 20 out of 41) were still concerned about the efficacy. Despite the majority of patients evaluating the safety and efficacy of biosimilars as equal to that of originators, there remains significant apprehension about biosimilar usage. Interestingly, this tendency was also observed among Korean oncologists, who, despite acknowledging the comparable safety and efficacy of biosimilars to originators, showed a stronger preference for originators and expressed concerns about switching to biosimilars due to safety and efficacy (Shin et al., 2024). Although people, in general, recognize the potential parity in safety and efficacy, they are still not convinced about the comparability of biosimilars in practical applications.

Secondly, patients exhibited similar patterns when evaluating the safety and efficacy of biosimilars. Korean patients perceived safety and efficacy as interconnected rather than separate issues. For instance, among the 41 respondents who believed the efficacy of biosimilars to be equivalent to that of originators, and among the 45 who assessed the safety of biosimilars as equivalent, a majority (N = 30) rated both safety and efficacy as equal to originators, suggesting that most respondents perceived these aspects similarly. This consistency may be due to insufficient comprehension of safety and efficacy, or patients may genuinely perceive safety and efficacy as equally important. This is where follow-up studies are needed.

The survey results regarding the price of biosimilars were noteworthy as well. The price of biosimilar products was the third most significant concern regarding biosimilars (N = 19), following safety and efficacy. 62% of patients indicated that biosimilars in Korea are expensive, while only 26% considered the price to be appropriate. It is notable that even the majority of high-income participants (26 out of 38), whose average monthly income is $6,000 USD or above—as defined by Korea’s Ministry of Economy and Finance—also agreed that biosimilars in Korea are overpriced. Indeed, a previous study has already shown biosimilars in Korea are generally more expensive compared to other countries (Kim et al., 2020). A previous study revealed that oncologists primarily prescribe biosimilars to alleviate patients’ financial burden (Shin et al., 2024); however, patients still find the price of biosimilars to be substantial.

Regarding biosimilar preference, a significant proportion of neutrality was observed among Korean patients. Specifically, 61% remained neutral towards biosimilar usage, while one-third (28%) were willing to use biosimilars and 11% not willing. Among the patients who exclusively used biosimilars, 95% (19 out of 20) reported that their doctor’s prescription and recommendation were the primary reasons for their treatment choice. Similarly, 91.7% of those who solely used originators (22 out of 24) cited the same reason for their treatment selection, and 58.3% (14 out of 24) of them indicated they would switch to biosimilars if their doctor recommended or prescribed biosimilars. This data suggests that patient preferences are heavily influenced by their doctor’s recommendations and prescriptions, highlighting the importance of encouraging physicians to prescribe biosimilars. Consequently, in addition to educating patients, it is crucial to implement usage-enhancing policies that motivate physicians to prescribe biosimilars in South Korea (Kim et al., 2020).

There are several limitations in this study. Firstly, the survey was conducted with patients at Ewha Womans University Medical Centers in Seoul. Due to nationwide disruptions in major hospitals caused by doctors’ strikes, distributing the survey was challenging, resulting in a relatively small number of respondents. Additionally, while our sample does not perfectly reflect the national age distribution, primarily due to an online survey format, the participants’ monthly income distribution resembles the national income distribution reported by Statistics Korea and the Survey of Household Finances and Living Conditions, indicating the representativeness of our survey sample at least in terms of economic status. Moreover, it should be noted that the educational level of participants, which could influence their understanding of biosimilars, was not considered in this survey. In addition, the survey relied on patients’ self-assessment of their knowledge and perception. For instance, instead of directly testing their understanding of biosimilars with specific questions, the survey asked how well they perceive biosimilars, following methodologies used in the previous studies (Jacobs et al., 2016; Macaluso et al., 2020). This approach may lead to slight discrepancies between the survey results and the actual level of patient awareness. Further studies considering these limitations are needed to enhance understanding of patient awareness. Nevertheless, this study offers critical insights into the current awareness and preferences regarding biosimilars among South Korean patients. The findings can serve as a foundation for strategies to increase biosimilar adoption, ultimately contributing to the accessibility of patients and the sustainability of the healthcare budget.

## 5 Conclusion

This study highlights that most patients were unfamiliar with biosimilars and had a neutral preference for biosimilars. The large proportion of neutrality suggests room for improvement in patients’ awareness. While many Korean patients perceive biosimilars as equivalent to originators in safety and efficacy, significant reservations remain due to limited knowledge. The results of this study can inform strategies to foster biosimilar adoption, ultimately supporting healthcare sustainability and improving patient access to affordable treatments.

## Data Availability

The datasets presented in this article are not readily available because the raw data collected are not publicly available due to privacy restriction, but are available upon reasonable request. Requests to access the datasets should be directed to sjbae@ewha.ac.kr.
