# Supplementary material for: Unaware and unpowered: evaluating patient perceptions and preferences of biosimilars in South Korea
Source: Front Pharmacol. 2025 Mar 14;16:1551451. doi: 10.3389/fphar.2025.1551451 (PMC11949777; doi:10.3389/fphar.2025.1551451)
Supplement: Supplementary file 1 [file DataSheet1.pdf]

Supplementary Figure 1. Flowchart of survey structure

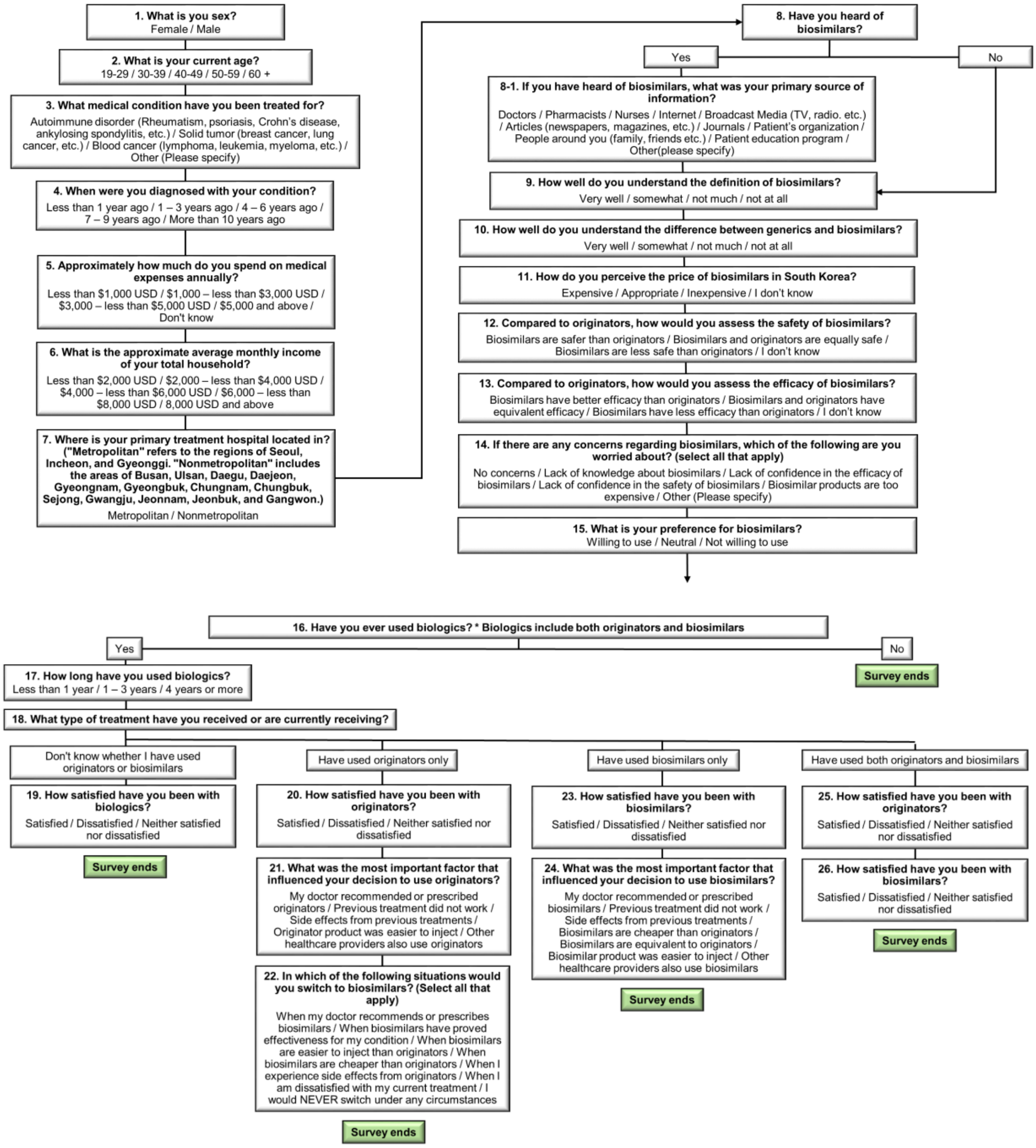

Supplementary Table 1. Checklist for Reporting Results of Internet E-Surveys (CHERRIES)

Item Category	Checklist item	Explanation	Page Number
Design	Describe survey design	Describe target population, sample frame. Is the sample a convenience sample? (In "open" surveys this is most likely.)	2 - 3
IRB (Institutional Review Board) approval and informed consent process	IRB approval	Mention whether the study has been approved by an IRB.	3
	Informed consent	Describe the informed consent process. Where were the participants told the length of time of the survey, which data were stored and where and for how long, who the investigator was, and the purpose of the study?	2 - 3 and p.8 of Supplementary Information
	Data protection	If any personal information was collected or stored, describe what mechanisms were used to protect unauthorized access.	N/A
Development and pre-testing	Development and testing	State how the survey was developed, including whether the usability and technical functionality of the electronic questionnaire had been tested before fielding the questionnaire.	2
Recruitment process and description of the sample having access to the questionnaire	Open survey versus closed survey	An "open survey" is a survey open for each visitor of a site, while a closed survey is only open to a sample which the investigator knows (password-protected survey).	2 - 3
	Contact mode	Indicate whether or not the initial contact with the potential participants was made on the Internet. (Investigators may also send out questionnaires by mail and allow for Web-based data entry.)	2 - 3

	Advertising the survey	How/where was the survey announced or advertised? Some examples are offline media (newspapers), or online (mailing lists – If yes, which ones?) or banner ads (Where were these banner ads posted and what did they look like?). It is important to know the wording of the announcement as it will heavily influence who chooses to participate. Ideally the survey announcement should be published as an appendix.	2 - 3 and p. 8 of supplementary Information
Survey administratio n	Web/E-mail	State the type of e-survey (eg, one posted on a Web site, or one sent out through e-mail). If it is an e-mail survey, were the responses entered manually into a database, or was there an automatic method for capturing responses?	2 - 3
	Context	Describe the Web site (for mailing list/newsgroup) in which the survey was posted. What is the Web site about, who is visiting it, what are visitors normally looking for? Discuss to what degree the content of the Web site could pre-select the sample or influence the results. For example, a survey about vaccination on an anti-immunization Web site will have different results from a Web survey conducted on a government Web Site	N/A
	Mandatory/vol untary	Was it a mandatory survey to be filled in by every visitor who wanted to enter the Web site, or was it a voluntary survey?	2 - 3
	Incentives	Were any incentives offered (eg, monetary, prizes, or non-monetary incentives such as an offer to provide the survey results)?	3 and p.8 of Supplementary Information

	Time/Date	In what timeframe were the data collected?	2 and p.8 of Supplementary Information
	Randomization of items or questionnaires	To prevent biases items can be randomized or alternated.	N/A
	Adaptive questioning	Use adaptive questioning (certain items, or only conditionally displayed based on responses to other items) to reduce number and complexity of the questions.	2
	Number of Items	What was the number of questionnaire items per page? The number of items is an important factor for the completion rate.	2
	Number of screens (pages)	Over how many pages was the questionnaire distributed? The number of items is an important factor for the completion rate.	2
	Completeness check	It is technically possible to do consistency or completeness checks before the questionnaire is submitted. Was this done, and if "yes", how (usually JAVAScript)? An alternative is to check for completeness after the questionnaire has been submitted (and highlight mandatory items). If this has been done, it should be reported. All items should provide a non-response option such as "not applicable" or "rather not say", and selection of one response option should be enforced.	3
	Review step	State whether respondents were able to review and change their answers (eg, through a Back button or a Review step which displays a summary of the responses and asks the respondents if they are correct).	3

Response rates	Unique site visitor	If you provide view rates or participation rates, you need to define how you determined a unique visitor. There are different techniques available, based on IP addresses or cookies or both.	N/A
	View rate (Ratio of unique survey visitors/unique site visitors)	Requires counting unique visitors to the first page of the survey, divided by the number of unique site visitors (not page views!). It is not unusual to have view rates of less than 0.1 % if the survey is voluntary.	N/A
	Participation rate (Ratio of unique visitors who agreed to participate/unique first survey page visitors)	Count the unique number of people who filled in the first survey page (or agreed to participate, for example by checking a checkbox), divided by visitors who visit the first page of the survey (or the informed consents page, if present). This can also be called "recruitment" rate.	N/A
	Completion rate (Ratio of users who finished the survey/users who agreed to participate)	The number of people submitting the last questionnaire page, divided by the number of people who agreed to participate (or submitted the first survey page). This is only relevant if there is a separate "informed consent"<br><br>page or if the survey goes over several pages. This is a measure for attrition. Note that "completion" can involve leaving questionnaire items blank. This is not a measure for how completely questionnaires were filled in. (If you need a measure for this, use the word "completeness rate".)	4
Preventing multiple entries from the same individual	Cookies used	Indicate whether cookies were used to assign a unique user identifier to each client computer. If so, mention the page on which the cookie was set and read, and how long the cookie was valid. Were duplicate entries avoided by preventing users access to the survey	Duplicate submissions were prevented by requiring participants to log in to a

		twice; or were duplicate database entries having the same user ID eliminated before analysis? In the latter case, which entries were kept for analysis (eg, the first entry or the most recent)?	Google account.
	IP check	Indicate whether the IP address of the client computer was used to identify potential duplicate entries from the same user. If so, mention the period of time for which no two entries from the same IP address were allowed (eg, 24 hours). Were duplicate entries avoided by preventing users with the same IP address access to the survey twice; or were duplicate database entries having the same IP address within a given period of time eliminated before analysis? If the latter, which entries were kept for analysis (eg, the first entry or the most recent)?	
	Log file analysis	Indicate whether other techniques to analyze the log file for identification of multiple entries were used. If so, please describe.	N/A
	Registration	In "closed" (non-open) surveys, users need to login first and it is easier to prevent duplicate entries from the same user. Describe how this was done. For example, was the survey never displayed a second time once the user had filled it in, or was the username stored together with the survey results and later eliminated? If the latter, which entries were kept for analysis (eg, the first entry or the most recent)?	Duplicate submissions were prevented by requiring participants to log in to a Google account.
Analysis	Handling of Incomplete questionnaires	Were only completed questionnaires analyzed? Were questionnaires which terminated early (where, for example, users did not go through all questionnaire pages) also analyzed?	Only complete responses were allowed to be submitted, ensuring there were no

			incomplete responses.
	Questionnaires submitted with an atypical timestamp	Some investigators may measure the time people needed to fill in a questionnaire and exclude questionnaires that were submitted too soon. Specify the timeframe that was used as a cut-off point, and describe how this point was determined.	N/A
	Statistical correction	Indicate whether any methods such as weighting of items or propensity scores have been used to adjust for the non-representative sample; if so, please describe the methods.	N/A

This checklist has been adapted from Eysenbach G. Improving the quality of Web surveys: the Checklist for Reporting Results of Internet E-Surveys (CHERRIES). J Med Internet Res. 2004 Sep 29;6(3):e34 [erratum in J Med Internet Res. 2012; 14(1): e8.]. Article available at https://www.jmir.org/2004/3/e34/; erratum available at https://www.jmir.org/2012/1/e8/.
Copyright ©Gunther Eysenbach. Originally published in the Journal of Medical Internet Research, 29.9.2004 and 04.01.2012

Supplementary Information. Survey Instrument Content

Welcome,

The Pharmacoeconomics, Policy and Outcomes Research Lab at Ewha Womans University, led by Professor SeungJin Bae, is conducting a survey titled "Online Survey Evaluating Patients' Perception on Biosimilar to Further Expand Biosimilar Market." This study aims to understand patient perceptions of biosimilars and collect insights to guide future policy-making and regulatory frameworks regarding biosimilars.

The survey consists of up to 26 questions and is expected to take approximately 10 minutes to complete. The survey will be conducted from November 2023 to August 2024. All responses will be anonymized and used exclusively for research purposes, with results potentially included in a published paper.

Your feedback is invaluable in shaping the development and refinement of policies and strategies related to biosimilars and biologics. We sincerely request your honest and comprehensive participation.

This survey is specifically designed for patients diagnosed with conditions treated with biologic injectables (excluding insulin products). As a token of our appreciation, participants who complete the survey will receive a coffee coupon.

For any further inquiries, please contact us at the details provided below.

Thank you for your participation.

※ Principal Investigator: Professor SeungJin Bae, College of Pharmacy, Ewha Womans University (sjbae@ewha.ac.kr)

※ Contact: Eunjung Choi, Master's Candidate, College of Pharmacy, Ewha Womans University (eunjung34@ewhain.net)

※ This survey contains three key terms. Please consider following terms carefully when responding:

Biologics: Biologics are high-molecular-weight medicines derived from living organisms including recombinant proteins, antibody drugs, vaccines, and gene therapies. Biologics include both originators and biosimilars.

Originator: An originator is the original biopharmaceutical product that was first developed and patented as a new therapeutic drug.

Biosimilar: A biosimilar is a biopharmaceutical drug designed to be highly similar to a patent-expired originator.

Survey Questionnaire

Please select the option that best describes your situation.

0. Do you agree to participate in the patient perception survey regarding biosimilars?

① I agree **[Survey begins]**

② I don’t agree **[Survey ends]**

Demographic Questions

1. What is your sex?
   ① Female
   ② Male
2. What is your current age?
   ① 19-29
   ② 30-39
   ③ 40-49
   ④ 50-59
   ⑤ 60 +
3. What medical condition have you been treated for?
   ① Autoimmune disorder (Rheumatism, psoriasis, Crohn’s disease, ankylosing spondylitis, etc.)
   ② Solid tumor (breast cancer, lung cancer, etc.)
   ③ Blood cancer (lymphoma, leukemia, myeloma, etc.)
   ④ Other (Please specify)
4. When were you diagnosed with your condition?
   ① Less than 1 year ago
   ② 1 – 3 years ago
   ③ 4 – 6 years ago
   ④ 7 – 9 years ago
   ⑤ More than 10 years ago
5. Approximately how much do you spend on medical expenses annually?
   ① Less than $1,000 USD
   ② $1,000 – less than $3,000 USD
   ③ $3,000 – less than $5,000 USD
   ④ $5,000 and above
   ⑤ Don't know
6. What is the approximate average monthly income of your total household?
   ① Less than $2,000 USD
   ② $2,000 – less than $4,000 USD
   ③ $4,000 – less than $6,000 USD
   ④ $6,000 – less than $8,000 USD
   ⑤ $8,000 USD and above

7. Where is your primary treatment hospital located in? ("Metropolitan" refers to the regions of Seoul, Incheon, and Gyeonggi. "Nonmetropolitan" includes the areas of Busan, Ulsan, Daegu, Daejeon, Gyeongnam, Gyeongbuk, Chungnam, Chungbuk, Sejong, Gwangju, Jeonnam, Jeonbuk, and Gangwon.)
   ① Metropolitan
   ② Nonmetropolitan

General Perceptions

8. Have you heard of biosimilars?
   ① Yes **[Move to Q8-1]**
   ② No **[Move to Q9]**

   8-1. If you have heard of biosimilars, what was your primary source of information?
   ① Doctors
   ② Pharmacists
   ③ Nurses
   ④ Internet
   ⑤ Broadcast Media (TV, radio. etc.)
   ⑥ Articles (newspapers, magazines, etc.)
   ⑦ Journals
   ⑧ Patient's organization
   ⑨ People around you (family, friends etc.)
   ⑩ Patient education program
   ⑪ Other (please specify)

9. How well do you understand the definition of biosimilars?
   ① Very well
   ② Somewhat
   ③ Not much
   ④ Not at all

10. How well do you understand the difference between generics and biosimilars?
   ① Very well
   ② Somewhat
   ③ Not much
   ④ Not at all

11. How do you perceive the price of biosimilars in South Korea?
   ① Expensive
   ② Appropriate

③ Inexpensive
④ I don’t know

12. Compared to originators, how would you assess the safety of biosimilars?
① Biosimilars are safer than originators
② Biosimilars and originators are equally safe
③ Biosimilars are less safe than originators
④ I don’t know

13. Compared to originators, how would you assess the efficacy of biosimilars?
① Biosimilars have better efficacy than originators
② Biosimilars and originators have equivalent efficacy
③ Biosimilars have less efficacy than originators
④ I don’t know

14. If there are any concerns regarding biosimilars, which of the following are you worried about? (Select all that apply)
① No concerns
② Lack of knowledge about biosimilars
③ Lack of confidence in the efficacy of biosimilars
④ Lack of confidence in the safety of biosimilars
⑤ Biosimilar products are too expensive
⑥ Other (Please specify)

15. What is your preference for biosimilars?
① Willing to use
② Neutral
③ Not willing to use

16. Have you ever used biologics? * Biologics include both originators and biosimilars
① Yes
② No **[Survey ends]**

17. How long have you used biologics?
① Less than 1 year
② 1-3 years
③ 4 years or more

18. What type of treatment have you received or are currently receiving?
① Don't know whether I have used originators or biosimilars **[Move to Q19]**
② Have used originators only **[Move to Q20]**
③ Have used biosimilars only **[Move to Q23]**
④ Have used both originators and biosimilars **[Move to Q25]**

Unsure Users

19. How satisfied have you been with biologics?
    ① Satisfied
    ② Dissatisfied
    ③ Neither satisfied nor dissatisfied

[Survey ends]

Originator only users

20. How satisfied have you been with originators?
    ① Satisfied
    ② Dissatisfied
    ③ Neither satisfied nor dissatisfied
21. What was the most important factor that influenced your decision to use originators?
    ① My doctor recommended or prescribed originators
    ② Previous treatment did not work
    ③ Side effects from previous treatments
    ④ Originator product was easier to inject
    ⑤ Other healthcare providers also use originators
22. In which of the following situations would you switch to biosimilars? (Select all that apply)
    ① When my doctor recommends or prescribes biosimilars
    ② When biosimilars have proved effectiveness for my condition
    ③ When biosimilars are easier to inject than originators
    ④ When biosimilars are cheaper than originators
    ⑤ When I experience side effects from originators
    ⑥ When I am dissatisfied with my current treatment
    ⑦ I would NEVER switch under any circumstances

[Survey ends]

Biosimilar only users

23. How satisfied have you been with biosimilars?
    ① Satisfied
    ② Dissatisfied
    ③ Neither satisfied nor dissatisfied
24. What was the most important factor that influenced your decision to use biosimilars?
    ① My doctor recommended or prescribed biosimilars

② Previous treatment did not work

③ Side effects from previous treatments

④ Biosimilars are cheaper than originators

⑤ Biosimilars are equivalent to originators

⑥ Biosimilar product was easier to inject

⑦ Other healthcare providers also use biosimilars

[Survey ends]

Both originator and biosimilar users

25. How satisfied have you been with originators?

① Satisfied

② Dissatisfied

③ Neither satisfied nor dissatisfied

26. How satisfied have you been with biosimilars?

① Satisfied

② Dissatisfied

③ Neither satisfied nor dissatisfied

[Survey ends]
